# Mitochondria in White, Brown, and Beige Adipocytes

**DOI:** 10.1155/2016/6067349

**Published:** 2016-03-17

**Authors:** Miroslava Cedikova, Michaela Kripnerová, Jana Dvorakova, Pavel Pitule, Martina Grundmanova, Vaclav Babuska, Dana Mullerova, Jitka Kuncova

**Affiliations:** ^1^Department of Physiology, Faculty of Medicine in Pilsen, Charles University in Prague, 301 00 Pilsen, Czech Republic; ^2^Biomedical Centre, Faculty of Medicine in Pilsen, Charles University in Prague, 301 00 Pilsen, Czech Republic; ^3^Department of Biology, Faculty of Medicine in Pilsen, Charles University in Prague, 301 00 Pilsen, Czech Republic; ^4^Department of Public Health and Preventive Medicine, Faculty of Medicine in Pilsen, Charles University in Prague, 301 00 Pilsen, Czech Republic; ^5^Department of Histology and Embryology, Faculty of Medicine in Pilsen, Charles University in Prague, 301 00 Pilsen, Czech Republic; ^6^Institute of Medical Chemistry and Biochemistry, Faculty of Medicine in Pilsen, Charles University in Prague, 301 00 Pilsen, Czech Republic

## Abstract

Mitochondria play a key role in energy metabolism in many tissues, including cardiac and skeletal muscle, brain, liver, and adipose tissue. Three types of adipose depots can be identified in mammals, commonly classified according to their colour appearance: the white (WAT), the brown (BAT), and the beige/brite/brown-like (bAT) adipose tissues. WAT is mainly involved in the storage and mobilization of energy and BAT is predominantly responsible for nonshivering thermogenesis. Recent data suggest that adipocyte mitochondria might play an important role in the development of obesity through defects in mitochondrial lipogenesis and lipolysis, regulation of adipocyte differentiation, apoptosis, production of oxygen radicals, efficiency of oxidative phosphorylation, and regulation of conversion of white adipocytes into brown-like adipocytes. This review summarizes the main characteristics of each adipose tissue subtype and describes morphological and functional modifications focusing on mitochondria and their activity in healthy and unhealthy adipocytes.

## 1. Introduction

Over the past few decades, the number of studies in the field of adipose tissue biology has increased exponentially since obesity and associated diseases are occurring at epidemic rates not only in developed countries, but also in developing countries. Obesity arises from an imbalance between energy intake and expenditure. It is associated with an increased risk of type 2 diabetes, hypertension, atherosclerosis, heart disease, stroke, cancer, infertility, and so forth [1–5]. Current clinical approaches to obesity include diet, physical activity, psychological support, drugs, and surgery treatment. Unfortunately, these treatment methods show efficiency limited only to small percentage of patients and some of them may be accompanied by serious side effects.

Studies published over the last two decades have established adipose tissue as a dynamic organ that carries out several important physiological processes. It is composed of a number of cell types: adipocytes, preadipocytes, vascular endothelial cells, pericytes, macrophages, and fibroblasts [6]. However, the dominant cells present in adipose tissue are mature adipocytes.

Two major types of adipose tissue exist in mammals, brown and white fat that have essentially antagonistic functions, brown fat expending energy and white fat storing it [7]. Brown adipocytes may occur after thermogenic stimulation at anatomical sites corresponding to WAT. This process is called the “browning” of WAT and these brown-like adipocytes that appear in WAT are called “beige” or “brite” [8, 9]. These three types of adipose cells have many specific characteristics related to localization, cell composition (lipid droplet, mitochondria), function, pathways of homeostatic control, obesity related changes, and so forth [8, 10].

Recent data suggest that adipocyte mitochondria might play an important role in the development of obesity through defects in mitochondrial lipogenesis and lipolysis, regulation of adipocyte differentiation, apoptosis, production of oxygen radicals, efficiency of oxidative phosphorylation, and regulation of conversion of white adipocytes into brown-like adipocytes [11, 12]. Thus, therapeutic intervention into any of these mitochondrial processes could be a useful approach to reduce adiposity [13].

This review summarizes the main characteristics of each adipose tissue subtype and describes morphological and functional modifications focusing on mitochondria and their activity in healthy and unhealthy adipocytes.

## 2. Overview of Mitochondrial Functions

Mitochondria are the cytoplasmic organelles in human and animal cells where many distinct metabolic pathways take place [14]. Mitochondria are highly dynamic, pleomorphic organelles comprising at least six compartments: outer membrane, inner boundary membrane of significantly larger surface area, intermembrane space, cristal membranes, intracristal space, and protein rich matrix. They are found in almost all human cells except mature erythrocytes [15, 16]. Although mitochondria contain their own small mtDNA and some RNA components of mitochondrial translational apparatus, the vast majority of the mitochondrial proteins are encoded by nuclear DNA, synthesized in the cytosol, and then imported into the mitochondria posttranscriptionally [15]. Mitochondria are involved in the crucial metabolic processes including tricarboxylic acid cycle, pyruvate decarboxylation, oxidative decarboxylation of fatty acids (*β*-oxidation), and degradation of branched amino acids. Mitochondria also substantially contribute to biosynthetic processes taking place in the cytosol by providing key intermediates like urea cycle, fatty acids, and heme synthesis. However, the principal role of mitochondria is to synthesize more than 95% of adenosine triphosphate (ATP) for cellular utilization [14, 16]. Production of ATP requires two major steps, oxidation of highly reducing metabolites and coenzymes such as nicotinamide adenine dinucleotide (NADH) and flavin adenine dinucleotide (FADH_2_) and phosphorylation of adenosine diphosphate to generate ATP to support various cellular functions (OXPHOS, oxidative phosphorylation) [17]. The mitochondrial respiratory system consists of four enzymatic multiheteromeric complexes (I–IV) embedded in the inner membrane of mitochondria and two individual mobile molecules, coenzyme Q (CoQ) and cytochrome* c*, along which the electrons liberated by the oxidation of NADH and FADH_2_ are passed and ultimately transferred to molecular oxygen. This respiratory process creates the electrochemical gradient of protons and membrane potential about 180 mV across the inner membrane that has the potential to do work. The proton flux drives the F_0_F_1_ ATP synthase (complex V) to phosphorylate matrix ADP by inorganic phosphate [18, 19]. On the other hand, mitochondria generate heat by a mechanism called “proton leak.” Protons leak from the intermembrane space to matrix and reduce membrane potential generating heat instead of energy [17]. Mitochondria are also deeply involved in the production of reactive oxygen species (ROS) through electron carriers in the respiratory chain. Oxidative stress can induce apoptotic death and mitochondria have a central role in this process due to cytochrome* c* release in the cytoplasm and opening of the permeability transition pore [20, 21]. Mitochondria are essential for the maintenance of normal physiological function of tissue cells and mitochondrial dysfunction may cause pathological changes in the human body [14]. In addition, eukaryotic cells have the ability to initiate adaptive responses to different environmental stimuli (e.g., cell growth, death and differentiation, or modification in energy demands) by altering the number, morphology, or remodelling of mitochondria [11].

## 3. White versus Brown versus Beige Adipocyte Tissue

**Figure 1:**
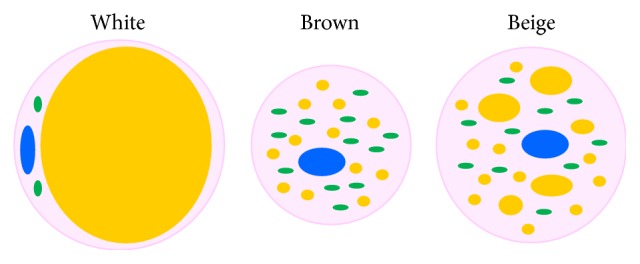
Main morphological characteristics of white, brown, and beige adipose tissues. White adipocyte cell is classically spherical, it is full of single lipid droplet, and it contains few mitochondria. Brown adipocyte is usually smaller than white and beige ones. It contains a large number of mitochondria and contains multiple small lipid droplets. Blue: nucleus, green: mitochondria, and yellow: lipid droplets.

In mammals, we can find three types of adipose depots commonly classified according to their colour appearance: the white (WAT), the brown (BAT), and the beige/brite/brown-like (bAT) adipose tissues. Main characteristics of WAT, BAT, and bAT in humans are shown in Table 1 and main morphological differences are shown in Figure 1. Most mammals have WAT dispersed throughout the body in two major types of depots, subcutaneous and intra-abdominal (or visceral). Intra-abdominal fat includes retroperitoneal, omental, mesenteric, epicardial, and gonadal deposits. WAT weight generally represents as much as 20% of the body weight of normal adult human and primarily acts as a storage site for triglycerides, conserving excess calories for use in times of scarcity. White adipocytes contribute to the whole body insulation and have endocrine functions including secretion of leptin, TNF-*α*, adiponectin, resistin, and other compounds related to the degree of obesity and insulin sensitivity [22].

Humans have relatively large depots of BAT in infancy; only small amounts of BAT dispersed throughout the depots of WAT persist in adults. Classical brown fat is primarily distributed in the interscapular space, paravertebrally, axillary, and perirenally. Recent studies have confirmed the presence of active BAT containing both classical brown and beige adipocytes in adult humans, with depots residing in the cervical, supraclavicular, mediastinal, paravertebral, and suprarenal regions [27, 39, 40].

White adipocyte cell is classically spherical and large with flattened nucleus, which is situated in the periphery. Because it is nearly completely filled with a single lipid droplet, thin ring of cytoplasm contains few mitochondria and little but recognizable smooth endoplasmic reticulum [41]. Brown adipocyte is usually smaller than the white one and its shape is elliptical with round or oval nucleus situated centrally. Cytoplasm volume is large containing multiple small lipid droplets, poor endoplasmic reticulum, and high amount of mitochondria.

**Figure 2:**
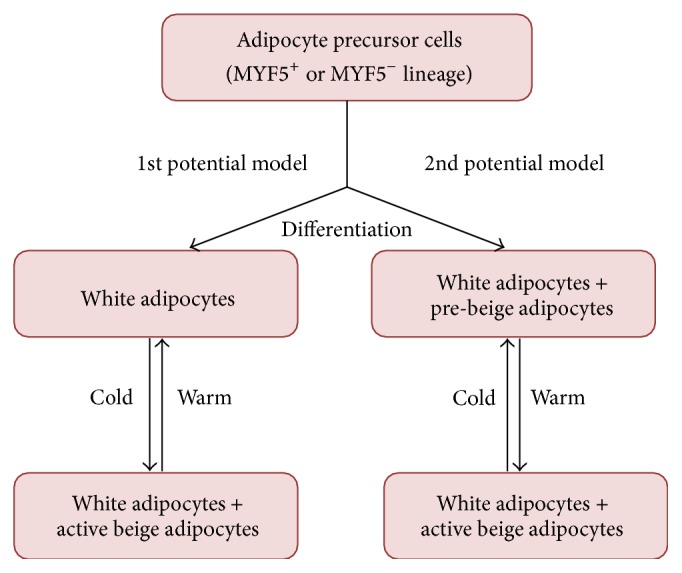
Two potential models of how WAT can be transformed into beige adipocytes [23, 25, 26].

Beige adipocyte has the mixed characteristics of both white and brown adipose cells. During basal state, it displays unilocular morphology as white adipocyte, but, upon cold stimulation, its appearance acquires features of intermediate morphology ultimately resulting in expression of proteins typical for BAT and transformation of stored fat into the small lipid droplets typical for brown adipocytes [8, 42, 43]. The origin and function of beige adipocytes are less clear and currently under intense discussion. It is thought that they arise from unique precursor cells [42], but there is also evidence that they stem from white adipocytes by transdifferentiation of preexisting white adipocytes. Himms-Hagen et al. treated rats with *β*3-adrenoceptor agonist (CL-316243); the results of their study showed that at least a subpopulation of unilocular adipocytes underwent conversion to multilocular mitochondria-rich adipocytes [44]. Interestingly, Morroni and coworkers suggested a new mechanism of reversible physiological transdifferentiation of adipocytes in the mammary gland: mouse mammary adipocytes are able to transform into secretory epithelial cells during pregnancy and revert to adipocytes after lactation [45]. Moreover, recent research has shown novel mechanism of the bAT formation. Wang et al. suggested that during cold-induced “browning” of subcutaneous fat, most “beige” adipose cells stem from* de novo* differentiated adipocytes [46]. Vargas et al. found that adipocytes differentiated with total and partial agonists of peroxisome proliferator-activated receptor gamma (PPAR*γ*) and exposed to 31°C are able to respond to cold by a significant increase in the expression of thermogenic proteins such as uncoupling protein 1 (UCP1), peroxisome proliferator-activated receptor c coactivator 1 (PGC1*α*), and Cbp/p300-interacting transactivator, with Glu/Asp-rich carboxy-terminal domain 1 (CITED1), a marker of the beige phenotype [28]. Two potential models of mature WAT into bAT transformation are shown in Figure 2.

Interestingly, exercise has been recently considered as a physiological stimulus for brown adipose tissue activity [47]. Even vibration training changed lipid metabolism in rats and promoted brown fat-like modifications in white adipose tissues through triggering BAT-associated gene expression, inflammatory response, and decrease in energy reserve [48]. Understanding these biological processes and stimulation of the activity of brown and beige/brite adipocytes could help us with fight against obesity, potentially facilitate weight loss, and improve metabolic health [49].

## 4. Mitochondrial Activity in Adipocytes

Mitochondria play a central role in metabolism of adipose tissue, as documented by their contribution to metabolic pathways of particular importance in adipocytes, like lipolysis and lipogenesis [11]. In addition, specific function performed by brown fat is converting mitochondrial energy into heat in adaptive thermogenesis. Tissue-specific functions of mitochondria in white fat are less characterized [50], although their role in orchestrating metabolic homeostasis and weight control is now widely accepted [51].

Lipolysis in adipocytes is the hydrolysis of triglycerides from lipid droplets within the cell into glycerol and free fatty acids by hormone-sensitive lipase (HSL) and adipose triglyceride lipase (ATGL). The hydrolytic action of HSL is regulated by perilipin A, a lipid droplet-associated protein. Phosphorylation of perilipin A by cAMP-dependent protein kinase (PKA) facilitates the translocation of HSL into the lipid droplet [52]. In the cytoplasm, free fatty acids are presumably bound to binding proteins and subsequently moved across the inner mitochondrial membrane by diffusion or, in the case of long carbon chains, by the carnitine shuttle [39, 53]. B-oxidation, metabolic process breaking down free fatty acids into acetyl-CoA takes place in the mitochondrial matrix. Acetyl-CoA then undergoes oxidation through the tricarboxylic acid cycle and the electron transport system.

The lipogenesis* de novo* is an important pathway to convert fatty acids to triglycerides for storage in the WAT. The human liver is mainly responsible for the conversion of carbohydrates into fatty acids, but a small part of triglycerides is synthesized in adipocytes [52]. Although fatty acids and triglycerides synthesis take place in the cytosol, mitochondria provide key intermediates needed for lipogenesis, like glycerol 3-phosphate and acetyl-CoA. Key enzyme in glycerol 3-phosphate synthesis is mitochondrial pyruvate carboxylase that converts pyruvate into oxaloacetate. Pyruvate also undergoes decarboxylation to acetyl-CoA by the mitochondrial pyruvate dehydrogenase complex, which facilitates fatty acid and triglyceride synthesis [54].

As mentioned above, brown/beige adipocytes, when activated by sympathetic stimulation, dissipate chemical energy stored in the form of triglycerides by channelling fatty acids into *β*-oxidation. Energy of substrate oxidation is then converted into heat [49, 55, 56]. This process, termed nonshivering thermogenesis, is specific function of BAT/bAT and is particularly important during hibernation and for small animals and infants who have greater demands on thermogenesis due to a large surface-to-volume ratio [57]. The molecular substrate of this unique function is a protein containing three similar repeats of about 100 amino acids coded by nuclear genes and inserted into the inner mitochondrial membrane [58]. As the major role of the protein is proton translocation resulting in uncoupling of the electron-transporting system from ATP synthesis in the mitochondria, it was named uncoupling protein (UCP) [59].

Uncoupling proteins belong to a family of mitochondrial carrier proteins that are present in the mitochondrial inner membrane. Mammals express five UCP homologues (UCP1 also named thermogenin), UCP2, UCP3, UCP4, and UCP5, also known as brain mitochondrial carrier protein 1 (BMCP1) [60]. UCP1 is expressed almost exclusively in fully differentiated BAT cells [17], although some findings suggest that UCP1 can be detected also in other tissues including uterine smooth muscle and even WAT, where induction of uncoupling protein expression is associated with acquiring of brown fat features [61, 62]. Expression of UCP1 in WAT has been questioned by finding of brown adipocytes in white depots and white adipocytes that potentially could transdifferentiate into cells expressing markers of BAT/bAT after appropriate stimulation [56, 62]. UCP2 and UCP3 mRNAs have been detected in a number of tissues and organs, for example, thymus, stomach, testis, white and brown adipocytes, pancreatic *β*-cells (UCP2) and skeletal muscle, heart, and brown adipocytes (UCP3) [63–67]. The physiological function of UCP1 is to mediate a regulated proton leak and thus dissipate the proton electrochemical gradient built up by the respiratory chain in the form of heat. Maximally stimulated brown adipose tissue can produce about 300 W/kg of heat compared to 1 W/kg in all other tissues [29, 68]. The thermogenesis in BAT is induced and positively regulated by fatty acids; in fact, no heat generation can be elicited without simultaneously initiating lipolysis. Further oxidation of acetyl-CoA the end product of *β*-oxidation, through tricarboxylic acid cycle and the electron transport chain, provides energy dissipated as heat through the action of UCP1 [69]. In contrast to UCP1, physiological function of its homologues is still debated. Recent studies have shown that UCPs might have an important role in pathogenesis of various disorders as type 2 diabetes, obesity, heart failure, neurodegenerative diseases, aging, or tumorigenesis [70–75].

Metabolic differences between mitochondria of WAT and BAT are associated with specific morphological characteristic of mitochondria in the brown adipocytes. These mitochondria are apparently more numerous and bigger in size and contain more cristae than mitochondria in white adipocytes. In addition, content of the heme cofactors in the mitochondrial enzyme cytochrome oxidase gives the tissue the brown macroscopic colour [16, 76]. Compared to BAT, WAT has fewer mitochondria, mostly undetectable expression of UCP1, and lower expression levels of the fatty acid *β*-oxidation-related enzyme, acyl CoA dehydrogenase, suggesting that the intensity of *β*-oxidation in WAT is lower than in BAT [53, 56, 77, 78]. As in other tissues, mitochondria represent the main source of ATP in the white fat. White fat mitochondria are well equipped for oxidative phosphorylation, with pyruvate serving as a main source of energy for ATP synthesis. Due to low activity of carnitine palmitoyltransferase 1 in the inner mitochondrial membrane, oxidation of fatty acids is relatively slow and fatty acids are directed towards esterification, unless the transferase is activated by leptin [79].

Forner et al. reported a systematic analysis of mouse mitochondrial proteomes of brown and white adipocytes documenting significant differences in the two sets of proteins, both qualitative and quantitative. Acetyl-CoA synthetase 2-like (gene* Acss1*), converting acetate to acetyl-CoA, and pyruvate dehydrogenase kinase 4 (gene* Pdk4*), inhibiting the pyruvate dehydrogenase complex thereby reducing the conversion of pyruvate to acetyl-CoA, were detected only in BAT. Conversely, MOSC domain-containing protein 1 (gene* Mosc1*), component of prodrug-converting complex, and acyl-coenzyme A synthetase ACSM5 (gene* Acsm5*), having CoA ligase activity, were detected only in WAT [50]. At transcript and proteome levels, BAT mitochondria were more similar to their counterparts in muscle cells. In contrast, WAT mitochondria not only selectively expressed proteins that support anabolic lipogenic function but also degrade xenobiotics and endogenous molecules, revealing a protective role of this tissue. These observations might help in better understanding of physiological processes in adipose tissue [50].

During adipocyte differentiation, the appropriate function of mitochondrion-specific metabolic processes is essential [11]. Adipogenic differentiation is characterized by the enhanced expression of some critical transcriptional factors, for example,* C/EBPa* and* PPARγ* [80, 81], lipid droplet accumulation, mitochondrial biogenesis [82], and a 20- to 30-fold increase in the concentration of numerous mitochondrial proteins [83]. ATP needed for the mitochondrial biogenesis, lipogenesis, and synthesis of numerous cytosolic and mitochondrial proteins is generated in the increased amounts due to the enhanced synthesis of mitochondrial DNA, subunits of respiratory complexes, cytochrome c, and enzymes of the tricarboxylic acid cycle [84]. In addition, tricarboxylic acid cycle generates citrate, which is then transported from the mitochondrion into the cytosol via the tricarboxylate carrier. Citrate is the only precursor of cytosolic acetyl-CoA, key intermediate used for fatty acid synthesis. Thus, citrate export from the mitochondria is essential during early differentiation stages of preadipocytes [85].

It has been also reported that a new adipose-specific protein, mouse ISG12b1, which is localized in the mitochondria, is predominantly overexpressed in adipocytes and dramatically induced at the terminal stage of adipogenesis. Functionally, ISG12b1 inhibits mitochondria biogenesis and adipocyte differentiation [86].

Taken together, although mitochondria in the brown fat are mainly acknowledged as important regulators of thermogenesis and those in the white fat as providers of constituents essential for lipogenesis, recent evidence suggests that mitochondria in adipose tissues might play plentiful roles in the regulation of the whole body energy homeostasis, crosstalk between adipose tissues and striated muscle, or control of insulin sensitivity and glucose metabolism [87–90].

## 5. Mitochondrial Dysfunction in Adipocyte

Mitochondrial dysfunction can result from a decrease in mitochondrial biogenesis, reduced mitochondrial content, and/or a decrease in the protein content and activity of oxidative proteins “per unit of mitochondria” [91]. The major tissues affected by mitochondrial dysfunction are those with a high energy demand such as heart, muscles, brain, and endocrine glands [11, 92]. However, in the past few years, many studies have targeted mitochondria in adipocytes or adipose tissues providing convincing evidence that impairment of mitochondrial functions in adipocytes could have the whole body pathological consequences [12, 51]. As mitochondria house crucial metabolic processes like fatty acid oxidation, oxidative phosphorylation, and ROS production, it is not surprising that impaired mitochondrial activity often has an association with metabolism and adipocyte differentiation [92].

### 5.1. Mitochondrial Dysfunctions in Metabolic Disorders

As shown in previous paragraphs, mitochondria contribute substantially to normal functions of adipose tissues. Although it is not clear yet if the mitochondrial dysfunction plays a causative or adaptive role in various metabolic disorders, further research in the field could reveal the correct timing of processes leading to obesity, insulin resistance, diabetes mellitus, or lipodystrophy. Compelling lines of evidence indicate that major factors contributing to mitochondrial defects in adipose tissues are (i) oxidative stress, (ii) insulin resistance, (iii) genetic factors, and also (iv) sedentary lifestyle without physical activity [93].

Oxidative stress is defined as a disturbance in the balance between the production of ROS and antioxidant defence [94]. Mitochondria are a major source of cellular free radicals that might damage proteins, lipids, and DNA. Defects in the transfer of electrons across the mitochondrial membrane can cause electrons to accumulate on the respiratory chain complexes, which results in an increase of the potential for electrons to bind with free oxygen and stimulation of ROS production [95]. Furukawa et al. have shown that elevated levels of fatty acids increased oxidative stress via NADPH oxidase activation in cultured adipocytes. ROS then caused dysregulated production of various adipocytokines, including adiponectin, plasminogen activator inhibitor-1, IL-6, and monocyte chemotactic protein 1 [96]. In obese mice, fat accumulation correlated with systemic oxidative stress and treatment with NADPH oxidase inhibitor reduced ROS production in adipose tissue, attenuated the dysregulation of adipocytokines, and improved diabetes, hyperlipidaemia, and hepatic steatosis in humans and mice [96]. Wang et al. have reported that higher intracellular ROS levels elicited by mitochondrial dysfunction resulted in the impairment of adipocyte function in the maintenance of glucose homeostasis through attenuation of insulin signalling, downregulation of the glucose transporter (*GLUT4*) expression, and decrease in adiponectin secretion [97].

Insulin resistance is a key defect associated with obesity and type 2 diabetes. It is defined as “a relative impairment in the ability of insulin to exert its effects on glucose, protein, and lipid metabolism in target tissues” [98]. Decreased insulin response to glucose, dyslipidaemia, and obesity frequently progress into overt type 2 diabetes with a decline in *β*-cell function, sustained hyperglycaemia, and increased advanced glycation end products (AGE) formation. In turn, AGE accumulation in adipose tissue may contribute to obesity-associated insulin resistance [99]. The role of mitochondria in adipose tissues in the onset and progression of insulin resistance is still a matter of controversy. Some recent findings suggest that dysregulation of mitochondrial calcium influx and efflux could be a crucial factor contributing to decreased insulin sensitivity [100] that is associated with impaired mitochondrial biogenesis and decreased expression of mitochondrial proteins in adipose tissues [101, 102]. However, mitochondrial dysfunction is not always essential for insulin resistance as reported by Martin et al. [103]. In addition, ROS-induced mitochondrial dysfunction seems to be a valid mechanism leading to insulin resistance in skeletal muscle, but not necessarily in adipocytes [103].

Genetic factors could play an important role in the onset and progression of obesity or type 2 diabetes mellitus. Among genes with positive associations of variants with obesity or obesity-related phenotypes, there are some deeply involved in the regulation of mitochondrial activity and biogenesis in adipose tissues, like* ADRB3* (adrenergic, *β*3 receptor),* INS* (insulin),* PLIN* (perilipin),* PPARγ* (peroxisome proliferative activated receptor, gamma), or* UCP1-UCP13* (uncoupling proteins 1–3) [104]. In addition, impaired expression of genes related to mitochondrial functions in adipose tissues can be caused by acquired mutations of both mitochondrial and nuclear genomes. Mitochondrial DNA displays a high mutation rate due to its specific features, like limited repair, proximity to ROS production, and absence of histones [105]. Defects in the expression of mitochondria-related genes were found at the mRNA as well as the protein levels in various organs and tissues including adipose cells [106–110].

Transcriptional coactivators PGC-1*α* and PGC-1*β* seem to be of particular importance in coordination of expression of mitochondrial and nuclear genes related to mitochondrial functions in both BAT and WAT [111, 112]. PGC-1*α* is able to direct human WAT PPAR*γ* toward a transcriptional program linked to energy dissipation through an increased expression of UCP1 [113]. Accordingly, decreased PGC-1*α* mRNA levels were reported in subcutaneous fat in morbidly obese subjects. Although it is not clear whether low PGC-1*α* expression is a prelude to the development of obesity or a consequence of it, upregulation of expression of thermogenic genes in white adipose tissue could offer new tool in the therapy of obesity [114].

However, it should be noted that impairment of expression of mitochondria-related genes does not necessarily lead to obesity as documented by manipulations with mitochondrial transcription factor A (TFAM), one of the major controllers of mitochondrial mass: in mice deficient in TFAM in adipocytes, activity of proteins in respiratory complexes I, III, and IV was severely compromised, which resulted in adipocyte death and inflammation in WAT and whitening of BAT [88].

Changes in human behaviour and lifestyle over the last century have resulted in a dramatic increase in the incidence of diabetes and obesity worldwide. Sedentary lifestyle, changes in work (from heavy labour to sedentary) have had an impact on human health [4, 115]. Physical activity is a major regulator of mitochondrial function in muscle cells and long-time inactivity is associated with reduced mitochondrial function and number [116].

In adipose tissues, regular physical activity and exercise training have long been known to cause increased expression and activity of mitochondrial proteins [117, 118]. In the last decade, the “beiging” of WAT associated with the expression of typical markers of BAT (like UCP1) in white adipocytes was revealed in response to exercise training [119, 120]. In rodents, even a single bout of exercise increased expression of a marker for mitochondrial biogenesis, PGC-1*α* mRNA in WAT [121]. This increase was presumably induced by stimulation *β*-adrenergic receptors at least in visceral WAT [122]. In the subcutaneous WAT, endothelial nitric oxide synthase has been proposed to regulate training-induced increases in mitochondrial biogenesis [118].

### 5.2. Mitochondrial Dysfunction during Adipocyte Differentiation

There is ample evidence that any damage to the mitochondrial respiratory chain results in compromised adipocyte differentiation. Inhibition of complex I by rotenone led to the significant reduction in the expression of mitochondrial malate dehydrogenase and a number of differentiation transcription factors, like PGC-1*β*, PPAR*γ*, CAAT/enhancer binding protein alpha (C/EBP*α*), and sterol regulatory element binding protein-1c (SREBP-1c). In addition, apparent decreases in the synthesis of triglycerides and ATP were reported [84]. Antimycin A, inhibitor of complex III, and oligomycin, inhibitor of ATP synthase commonly used for developing of mitochondrial dysfunction models, also prevented preadipocyte differentiation [123, 124].

High concentrations of mitochondrial ROS generated by the respiratory chain have also detrimental influence on adipoblast proliferation and differentiation. Genetic manipulation of mitochondrial complex III revealed that ROS generated by this complex were required to initiate primary human mesenchymal stem cells differentiation [81]. Thus, the production of ROS and mitochondrial metabolism are not simply a consequence of adipogenesis but are causal factors in promoting adipocyte differentiation [81].

## 6. Conclusions

Adipose tissue is an extremely plastic organ capable of massive expansion, reduction, or transformation according to appropriate stimulation. Research motivated mainly by the desire to understand adipocytes in the context of obesity and related diseases resulted not only in promising data opening new ways to fight obesity, but also in the discovery of multipotent stem cells within WAT [125]. It is now widely accepted that adipose tissue acts not only as repository for excess nutrients but also as integrator and regulator of the balance between food intake and energy output. It secretes a number of substances affecting the function of several organs in the body and also the function of adipose tissue itself [51, 126, 127]. This review summarizes the main characteristics of each adipose tissue subtype and describes morphological and functional modifications focusing on mitochondria and their activity in healthy and unhealthy adipocytes. Increasing evidence in adipocyte-related mitochondrial research demonstrates the important role of mitochondria in the onset or progression of obesity and related pathologies and offers a large spectrum of potential therapeutic targets, like differentiation and transformation of adipocytes, ROS production, substrate channelling to energy dissipation, or changes in the lifestyle.

## Figures and Tables

**Table 1:** Main differences amongst the three types of adipocyte tissue.

Characteristic property	White	Brown	Beige	References
Morphology				
(i) Shape	Spherical	Ellipsoid/polygonal	Spherical	[8, 9, 23]
(ii) Cell size	Variable, large (25–200 *µ*m)	Small (15–60 *µ*m)	Variable, smaller than white	
(iii) Lipid droplet (LD)	Single large LD	Multiple small LD	Multiple LD with variable size	
(iv) Mitochondria	+	+++	++ (upon stimulation)	

Development	From Myf5^−^ or Myf5^+^ precursors	From Myf5^+^ precursors	From Myf5^−^ or Myf5^+^ precursors	[23–26]

Location	Subcutaneous and visceral	Suprarenal, paravertebral, supraclavicular	Inguinal, neck (near carotid sheath and musculus longus colli), other locations?	[8, 27]

Function	Energy storage	Heat production	Adaptive thermogenesis	[9]

Uncoupling protein	Nearly undetectable	+++	++ (upon stimulation)	[28, 29]

Adipocyte-type-specific markers	*PPARγ, PLIN1, HOXC9, TCF21, TLE3, C/EBPα, Rb, Rip140*	*LHX8, ZIC1, EPSTI1, PRDM16, CIDEA, ELOVL3*	*HOXC8, HOXC9, CITED1, CD137, TMEM26, TBX1, CD40*	[13, 23, 30, 31]

Vascularization	Low	High	High upon stimulation	[32, 33]

Impact on obesity	Positive	Negative	Negative	[34]

Correlation with insulin resistance	Yes	Probably yes	Probably yes	[35–38]

CD40: CD40 molecule, TNF receptor superfamily member 5; CD137: tumour necrosis factor receptor superfamily, member 9; C/EBP*α*: CCAAT/enhancer binding protein (C/EBP), alpha; CIDEA: cell death-inducing DFFA-like effector; CITED1: Cbp/p300-interacting transactivator, with Glu/Asp-rich carboxy-terminal domain 1; ELOVL3: ELOVL fatty acid elongase 3; EPSTI1: epithelial stromal interaction 1; HOXC8: homeobox C8; HOXC9: homeobox C9; LHX8: LIM homeobox protein 8; PLIN1: perilipin-1; PPAR*γ*: peroxisome proliferator-activated receptor gamma; PRDM16: PR domain containing 16; Rb (Rb1): retinoblastoma 1; Rip140: nuclear receptor interacting protein 1; TBX1: T-Box 1; TCF21: transcription factor 21; TLE3: transducin-like enhancer of split 3; TMEM26: transmembrane protein 26; ZIC1: zinc finger protein of the cerebellum 1.
